# Effect of Comorbidity Assessed by the Charlson Comorbidity Index on the Length of Stay and Mortality Among Immobile Hemorrhagic Stroke Patients Younger Than 50 Years

**DOI:** 10.3389/fneur.2020.00487

**Published:** 2020-06-18

**Authors:** Hongpeng Liu, Xinjuan Wu, Jing Cao, Jing Jiao, Chen Zhu, Baoyun Song, Jingfen Jin, Yilan Liu, Xianxiu Wen, Shouzhen Cheng

**Affiliations:** ^1^Department of Nursing, Chinese Academy of Medical Sciences - Peking Union Medical College, Peking Union Medical College Hospital, Beijing, China; ^2^Department of Nursing, Henan Provincial People's Hospital, Zhengzhou, China; ^3^The Second Affiliated Hospital, Zhejiang University School of Medicine, Hangzhou, China; ^4^Department of Nursing, Wuhan Union Hospital, Wuhan, China; ^5^Department of Nursing, Sichuan Provincial People's Hospital, Chengdu, China; ^6^Department of Nursing, The First Affiliated Hospital, Sun Yat-sen University, Guangzhou, China

**Keywords:** hemorrhagic stroke, cerebrovascular disease, comorbidity, mortality, length of stay, young, Chinese

## Abstract

**Objective:** The burden of comorbidity among young patients with hemorrhagic stroke is high. This study examines the effect of comorbidity on the length of stay (LoS) and mortality among immobile hemorrhagic stroke patients younger than 50 years.

**Materials and Methods:** A retrospective *post-hoc* analysis study design was used. A total of 767 younger adults (mean age 41.64 ± 8.16 years) hospitalized for hemorrhagic stroke between November 2015 and July 2017 were evaluated. All-cause mortality was recorded for 90 days after admission regardless of whether death occurred before or after discharge. Comorbidity was assessed using the Charlson Comorbidity Index (CCI), categorized as low (0–1) and high (≥2). A multiple linear regression model was applied to examine the association between CCI and LoS. Survival was evaluated with Kaplan–Meier and Cox regression analyses.

**Results:** The mean CCI of all patients was 1.25 (SD ± 0.79) and median was 1.0 (IQR 1, 1). The average LoS for patients with a low CCI was 17.73 (± 11.59) days compared with 19.49 (± 15.21) days in those with a high CCI (*p* = 0.142). The mortality rate was 6.0% (12.10% for high CCI vs. 4.82% for low CCI, *p* = 0.002). After controlling for confounders, high CCI was not associated with longer LoS (regression coefficient 0.825, 95% CI −1.155 to 2.805; *p* = 0.413), whereas high CCI was associated with greater likelihood of death than a low CCI (hazard ratio 3.417, 95% CI 1.626 to 7.180; *p* = 0.001).

**Conclusions:** High global comorbidity was associated with increased short-term mortality but not with LoS. Measures to manage comorbidities aimed at reducing negative clinical impacts of stroke among younger adults are warranted.

## Introduction

Stroke is a major cause of death worldwide (1). In China, with a population of 1.4 billion, the annual mortality rate from stroke is approximately 1.6 million, i.e., 1 in 637, meaning that stroke has become the leading cause of death (2). Previous studies indicated that approximately two-fifths of all stroke deaths are attributable to hemorrhagic stroke (3). Compared with the United States and Europe, the incidence of hemorrhagic stroke is higher in Asia (4, 5), and the prevalence of hemorrhagic stroke in China is as high as 23.8% (6).

Previous studies have suggested an upward trend of stroke in younger people and a declining incidence of stroke in older adults (7–9). Stroke in younger adults has serious consequences. Those younger than 50 years of age are typically vocationally active. Death or disability attributable to stroke leads to a tremendous economic burden on society as well as patients themselves and their families (9–12). Several lines of evidence have suggested that mortality among younger stroke patients is lower than in older adults but significantly higher than in the age-adjusted general population (13, 14).

In addition, China has 7.5 million stroke survivors who suffer greatly through comorbidity (2). It was reported that approximately nine out of ten stroke patients have at least one comorbid condition and almost one-quarter have five or more comorbidities (15, 16). Stroke accompanied by comorbidities can directly or indirectly result in increasing disability or immobility (17, 18), thus inducing other medical complications and increasing the risk of Length of Stay in hospital (LoS), stroke recurrence, or even death (14, 19, 20). The impacts of comorbidities on survival among stroke patients aged 65 years and older have been identified (21–23). However, to our knowledge, studies of the effect of comorbidities on hemorrhagic stroke patients younger than 50 years are scarce in China.

This study was derived from a research project from the National Health and Family Planning Commission of the People's Republic of China, which aimed to develop a standardized nursing intervention model (SNIM) among immobile stroke patients. As one of the main gauges of this study, the Charlson Comorbidity Index (CCI) addresses multiple comorbidities by creating a weighted total score based on the presence of various conditions, and is commonly used in outcome and mortality studies (17, 22, 24). As part of the baseline survey for this large-scale prospective study, we carried out the present study to investigate whether a higher comorbidity burden (measured by CCI) is associated with increased short-term mortality and LoS among immobile sufferers of hemorrhagic stroke younger than 50 years of age.

## Materials and Methods

### Population and Design Study

Study subjects enrolled were the participants of the SNIM, a prospective study conducted in 25 general hospitals covering five provinces and one municipality city in China (Beijing municipality city, Henan province, Zhejiang province, Guangdong province, Hubei province, and Sichuan province). Further information about the SNIM study is published elsewhere (25). A total of 8,058 participants aged 18 years or older consecutively enrolled in the SNIM study between November 2015 and July 2017.

All 8,058 cases of stroke were defined by principal diagnosis codes I60.x, I61.x, H34.1, I63.x, I64.x, and G45.x according to the *International Classification of Disease, 10th revision*, and were assessed after dividing the cases into hemorrhagic stroke (I60.x, I61.x), acute ischemic stroke (AIS; H34.1, I63.x, I64.x), and transient ischemic attack (TIA; G45.x) (26). According to pathological subtypes, (1) I61.x represents intracerebral hemorrhage and I60.x represents subarachnoid hemorrhage.

All participants in SNIM, except for those who died or relinquished medical treatment, underwent a 90-day follow-up after enrollment in the study. All-cause mortality was recorded for 90 days including in-hospital deaths. Survival time was regarded as the duration from the date of admission to the date of death. Dates of death were collected from death certificates.

Comorbidity was assessed using the CCI. The index contained 19 comorbid conditions and was adjusted specifically for stroke evaluation (22, 23, 27). A CCI score of 0–1 indicates low comorbidity and a score of 2 or higher is considered high comorbidity (17, 21, 23, 28), unless otherwise specified. Comorbid conditions were recorded if present in the medical record.

In this study we considered a sample of participants with hemorrhagic stroke who were younger than 50 years (*N* = 767) because our aim was to evaluate young immobile hemorrhagic stroke patients with comorbidities, with the study outcomes focusing on mortality and LoS. Immobility was defined as when the patient's basic physiological needs were carried out in bed, except for active or passive bedside sitting/standing/wheelchair use for examination. LoS was defined as the period from the date of admission to the date of discharge or date of death. All-cause mortality was recorded for 90 days after the date of admission regardless of whether death occurred before or after discharge. Therefore, we opted to exclude participants in accordance with the following criteria: older than 50 years (*n* = 6,597), AIS (H34.1, I63.x, I64.x) cases (*n* = 623), TIA cases (*n* = 57), and discharged within 24 h of admission (*n* = 14).

This study was ethically approved by the review board of Peking Union Medical College Hospital. Patients enrolled gave their informed consent to participate in the study. Patients' records and information were anonymized and de-identified prior to the analysis.

### Definition of Covariates and CCI

Potential associated factors of mortality and LoS in the models included age, sex, ethnicity, education, tobacco smoking, experience of intensive care unit (ICU), experience of surgery, duration of immobility, level of hospital, payment type, and complications that occurred during hospital stay including pressure injuries, pneumonia, deep vein thrombosis (DVT), and urinary tract infections.

We assessed 19 diagnoses for each patient according to the CCI: “history of myocardial infarction (weight = 1),” “congestive heart failure (weight = 1),” “cerebrovascular disease (with mild or no residua or TIA, weight = 1),” “peripheral disease (including aortic aneurysm ≥6 cm, weight = 1),” “chronic pulmonary disease (weight = 1),” “connective tissue disease (weight = 1),” “peptic ulcer disease (weight = 1),” “dementia (weight = 1),” “mild liver disease (no portal hypertension, including chronic hepatitis, weight = 1),” “diabetes without end-organ damage (excluding diet-controlled alone, weight = 1),” “moderate or severe renal disease (weight = 2),” “diabetes with end-organ damage (retinopathy, neuropathy, nephropathy, or brittle diabetes, weight = 2),” “hemiplegia (weight = 2),” “tumor without metastasis (excluded if >5 years from diagnosis, weight = 2),” “leukemia (acute or chronic, weight = 2),” “lymphoma (weight = 2),” “moderate or severe liver disease (weight = 3),” “metastatic solid tumor (weight = 6),” and “AIDS (not just HIV positive, weight = 6)” (22, 28).

### Statistical Analyses

We employed descriptive statistics to summarize patients' characteristics. Variables were compared across patients in the two CCI categories using the χ^2^ test for proportions and Student's *t*-test for means. We used Kaplan–Meier survival curves and the log-rank test to compare mortality between the two groups. Sociodemographic and clinical parameters were adjusted by a Cox proportional hazards model. A multiple linear regression model was applied to evaluate the relationship between CCI and LoS while controlling for the above covariates listed. In all analyses, CCI was categorized as low (0–1) and high (≥2) comorbidity (29). A *p* < 0.05 indicated statistical significance. Statistical analysis was conducted in STATA software (version 15/IC; Stata Corp, College Station, TX, USA).

## Results

### Demographics

A total of 767 immobile hemorrhagic stroke patients younger than 50 years were hospitalized between November 2015 and July 2017. The mean age of the patients was 41.64 (standard deviation [SD] ± 8.16) years. The mean CCI of the patients was 1.25 (SD ± 0.79) and the median was 1.0 (interquartile range [IQR] 1, 1). The percentage of patients with high CCI score (≥2) was 16.17%. Forty-six patients died, and the average LoS was 18.01 (SD ± 12.26) days. Table 1 summarizes the patients' sociodemographic and clinical characteristics according to low- or high-CCI groups. Both groups were similar in most characteristics except that the low-CCI group experienced more surgery and pneumonia during hospital stay and contained more patients who belonged to The Public Health Insurance Program according to the payment type, while the high-CCI group experienced more ICU and DVT during hospitalization and contained more patients with a higher educational background and more smokers.

**Table 1:** Sociodemographic and clinical characteristics of the patients (*N* = 767).

**Characteristics**	**Charlson comorbidity index^§^**		
	**Low comorbidity (*N* = 643)**	**High comorbidity (*N* = 124)**	***p*-Value**
Mean age, years (*SD*)	41.390 (8.30)	42.927 (7.26)	0.055
Male, *n* (%)	370 (57.54)	83 (66.94)	0.051
Han ethic, *n* (%)	635 (98.76)	122 (98.39)	0.740
Education, *n* (%)			0.046*
University and above	105 (16.33)	33 (26.61)	
High school	100 (15.55)	16 (12.90)	
Junior high school	259 (40.28)	41 (33.06)	
Primary school	179 (27.84)	34 (27.42)	
Smoking, *n* (%)			0.032*
Non-smoker	431 (67.03)	78 (62.90)	
Current smoker	23 (3.58)	11 (8.87)	
Former smoker	189 (29.39)	35 (28.23)	
Experience of ICU, *n* (%)	66 (10.26)	22 (17.74)	0.017*
Experience of surgery, *n* (%)	244 (37.95)	35 (28.23)	0.039*
Duration of immobility, *n* (%)			0.460
1–3 days	38 (5.91)	8 (6.45)	
4–6 days	99 (15.40)	16 (12.90)	
7–12 days	209 (32.50)	49 (39.52)	
13 days and above	297 (46.19)	51 (41.13)	
Tertiary hospital, *n* (%)	564 (87.71)	107 (86.29)	0.661
Payment type, *n* (%)			0.015*
NCMS	241 (37.48)	29 (23.39)	
URBMI	61 (9.49)	12 (9.68)	
UEBMI	83 (12.91)	24 (19.35)	
The public health insurance program	11 (1.71)	0	
Self-paying	243 (37.79)	57 (45.97)	
Others	4 (0.62)	2 (1.61)	
PIs, *n* (%)	20 (3.11)	8 (6.45)	0.069
DVT, *n* (%)	3 (0.47)	6 (4.84)	<0.001**
Pneumonia, *n* (%)	111 (17.26)	38 (30.65)	0.001*
UTIs, *n* (%)	15 (2.33)	4 (3.23)	0.558
Death, *n* (%)	31 (4.82)	15 (12.10)	0.002*
Mean LoS, days (SD)	17.73 (11.59)	19.49 (15.21)	0.142

*p < 0.05, **p < 0.001.

§ *Low comorbidity (0–1), high comorbidity (≥2)*.

*CCI, Charlson Comorbidity Index; NCMS, New Cooperative Medical System (created to support rural residents); URBMI, Urban Resident Basic Medical Insurance (built to support urban residents without a stable job); UEBMI, Urban Employee Basic Medical Insurance (created to support employed workers); The Public Health Insurance Program provides payments for retired officials (who started work before 1949), civil servants, and government-affiliated employees; Self-paying means patients pay all of their own hospital fees. Others includes private commercial insurance companies that subsidize basic insurance coverage; PIs, pressure injuries; DVT, deep vein thrombosis; UTIs, urinary tract infections; SD, standard deviation; LoS, length of stay*.

### Length of Stay

Table 1 demonstrates LoS among the study participants. The average LoS in the low-CCI group (0–1) was 17.73 (SD ± 11.59) days compared with 19.49 (SD ± 15.21) days in the high-CCI group (≥2) (*p* = 0.142). As shown in Table 2, there was no significant difference between the high-comorbidity group and the LoS in the crude estimate analysis compared with the low-CCI group. Similar to the crude estimate analysis results, after adjusting for potential confounders in the multiple linear regression model, the association of the high-comorbidity group with LoS was not significant in the adjusted analysis (regression coefficient 0.825, 95% confidence interval [CI] −1.155 to 2.805; *p* = 0.413).

**Table 2:** Regression models of LoS and survival in high-comorbidity vs. low-comorbidity patients.

	**LoS**			**Mortality**		
	**Regression coefficient^b^**	**95% CI**	***p*-Value**	**Hazard ratio^c^**	**95% CI**	***p*-Value**
**Crude estimate**						
Low (Ref.)	–	–		–	–	
High	1.766	(−0.592, 4.124)	0.142	2.602	(1.405, 4.820)	0.002
**Adjusted^a^**						
Low (Ref.)	–	–		–	–	
High	0.825	(−1.155, 2.805)	0.413	3.417	(1.626, 7.180)	0.001

*CI, confidence interval; LoS, length of stay; Low, low comorbidity (0–1); High, high comorbidity (≥2)*.

a *Adjusted for duration of immobility, deep vein thrombosis, level of hospital, education, pneumonia, pressure injuries, experience of ICU, urinary tract infections, insurance, age*.

b *Multiple linear regression model*.

c *Cox proportional hazard model*.

### Mortality

**Figure 1:**
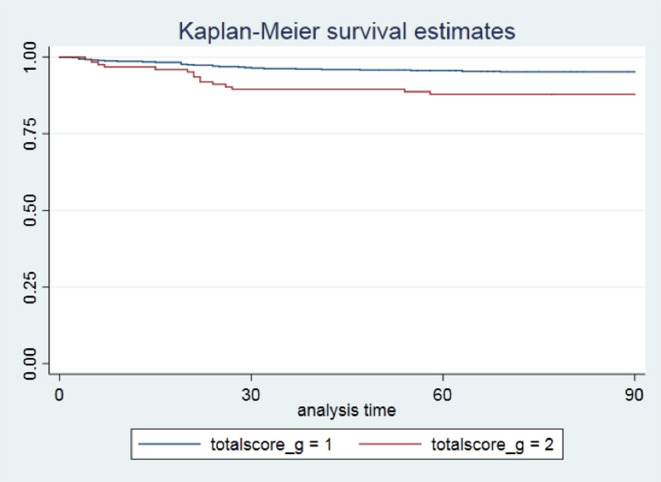
Unadjusted Kaplan–Meier survival curves for patients stratified by the Charlson Comorbidity Index. Log-rank test, *p* = 0.0016. CCI, Charlson Comorbidity Index; totalscore_g = 1, low comorbidity (0–1); totalscore_g = 2, high comorbidity (≥2).

Overall, death occurred in 6.0% of the study population. The number of patients with a low (0–1) and a high (≥2) CCI score who died were 31 (4.82%) and 15 (12.10%), respectively (*p* = 0.002) (Table 1). Patients in the low-CCI group had better survival rates than patients with a high comorbidity (*p* value for rank test = 0.0016) (Figure 1). As shown in Table 2, a high CCI score (≥2) was positively correlated with an approximately 2.6-fold higher risk of death than a low CCI score (0–1) (hazard ratio [HR] 2.602, 95% CI 1.405 to 4.820; *p* = 0.002) in the crude estimate analysis. In the Cox proportional hazards model, a high CCI score (≥2) was associated with an even greater likelihood of death compared with a low CCI score (0–1) after adjusting for confounders (HR 3.417, 95% CI 1.626 to 7.180; *p* = 0.001).

## Discussion

To our knowledge, this is the first study from China to focus on the effect of comorbidity on LoS and short-term mortality among immobile patients younger than 50 years of age with hemorrhagic stroke. The results indicated that a high CCI score (≥2) could be an effective predictor for higher risk of death compared with a low CCI score, although the present study did not show a significant association between increased LoS and a high CCI score. These findings highlight the essential clinical impact of comorbid conditions among a diverse population of younger patients from a developing country with a universal healthcare system.

No significant associations were found between LoS and comorbidities in this study, suggesting that this predictor of clinical outcome has less influence on LoS among the younger hemorrhagic stroke population. Contrary to our results, Richard et al. (21) analyzed the effect of comorbidity on LoS in a cohort of 776 older patients (mean age 80.1 ± 8.3 years) affected by stroke, whereby the mean CCI of the cohort was 2.21 (SD ± 2.2) and the median was 2.0 (IQR 0, 3). In addition, other studies suggested that there is a significant difference in CCI scores in relation to the age of hemorrhagic stroke patients, which may lead to different clinical outcomes (22, 30). However, the average CCI of the young (mean age 41.64 ± 8.16 years) hemorrhagic stroke population in this study was 1.25 (SD ± 0.79) and the median was 1.0 (IQR 1, 1), which was lower than that reported in a previous study (21). Therefore, this observation can be mainly justified by a difference in age related to a young sample population and a low CCI score.

In the present study we found a low mortality rate of 6.0%, which is lower than the previous studies reporting case fatality rates ranging from 7.7 to 9.9% among younger hemorrhagic and ischemic stroke patients (14, 31). However, the mortality rate in our study is higher than the rate of 4.5% in a study of ischemic stroke in young patients (32). These differences are likely due to a variety of factors, including subtypes of stroke, study design, enrollment of participants, and local factors (e.g., the standard of nursing care or medical care received), but mainly because all patients in the other studies are ischemic stroke sufferers (32), for which the mortality was significantly lower than that for hemorrhagic stroke (32–34).

Previous studies have reported an association between comorbidity and an increased rate of adverse clinical outcomes, particularly regarding mortality (22, 35, 36). Our finding that high comorbidity as measured by the CCI is associated with mortality among hemorrhagic stroke patients younger than 50 is in line with a previous study evaluating the validity of the CCI in assessing comorbidity and clinical outcome in stroke patients (22, 30). It further verified the use of the CCI as a valid method of evaluating comorbidities in younger stroke patients (18, 21, 22, 35) and testified to the use of CCI as a tool in predicting short-term death in the young hemorrhagic stroke population.

Comorbidities can hinder the young patient's recovery from a stroke (18). Therefore, addressing comorbidities is critical and should be considered as part of a broader strategy to reduce the adverse clinical effects of hemorrhagic stroke. In particular, understanding that mortality is closely related to stroke and concomitant comorbidities would help to better care for the stroke patient by placing greater emphasis on treating comorbidities instead of solely stroke (37). Furthermore, improved healthcare collaboration, interdisciplinary care transition, and management counseling in hospital are all important measures that could contribute to the optimized management of comorbidities and distribution of resources for stroke management (21, 38, 39). In short, a detailed assessment and management of the association between CCI and hemorrhagic stroke among younger adults requires further study.

Our study has some strengths. This study firstly demonstrated the prognostic value of comorbidity using the CCI for short-term survival among immobile hemorrhagic stroke patients younger than 50 years in China, whereby our findings reinforce the importance of monitoring chronic comorbidities. Besides this, in the future we will address long-term mortality, allowing us to better delineate a stroke-management scenario that to date has been lacking in the developing world.

As for limitations, firstly this study is a retrospective *post-hoc* analysis, whereby the data were prospectively collected for the SNIM research project and later were used retrospectively as part of our study design. Secondly, there is a lack of other relevant clinical data, including clinical severity of stroke (NIHSS), premorbid Rankin scale, volume of intracerebral bleeding, laboratory data, and the details of causes of death, because these features were not recorded in the first place, which is an inherent drawback of a retrospective analysis of a prospectively collected database. The data about the etiologic classification of patients with intracerebral hemorrhage, such as hypertension, and oral anticoagulants is limited. In addition, no affirmative criteria were available to determine the severity of comorbidities, which may potentially influence the clinical outcome of the participants. Prospective studies with more sophisticated evaluations are required to confirm our findings.

## Conclusion

Among hemorrhagic stroke patients younger than 50 years of age, patients in the high-CCI group (CCI ≥2) were associated with increased short-term mortality but not with LoS. More emphasis should be placed on comorbidities as part of wider strategies to reduce the negative clinical impact in young hemorrhagic stroke patients.

## Data Availability Statement

The datasets used and analyzed during the current study are available from the corresponding author on reasonable request.

## Ethics Statement

The studies involving human participants were reviewed and approved by the ethics committee of Peking Union Medical College Hospital. Patients' records and information were anonymized and de-identified prior to the analysis. The patients/participants provided their written informed consent to participate in this study.

## Author Contributions

XWu: study concept and design. XWu and HL: analysis and interpretation of data. HL: edited the manuscript and drafted the tables. JC and JJia: critical review of the manuscript for important intellectual content. CZ, BS, JJin, YL, XWe, and SC: recruited patients, collected data, and edited the manuscript. All authors critically reviewed and approved the manuscript before it was submitted.

## Conflict of Interest

The authors declare that the research was conducted in the absence of any commercial or financial relationships that could be construed as a potential conflict of interest.
